# Crosstalk between ILC2s and Th2 CD4^+^ T Cells in Lung Disease

**DOI:** 10.1155/2022/8871037

**Published:** 2022-05-10

**Authors:** Lan-lan Mi, Wei-wei Guo

**Affiliations:** Department of Neonatology, Shanghai Children's Medical Center, Shanghai Jiao Tong University School of Medicine, Shanghai, China

## Abstract

Cytokine secretion, such as interleukin-4 (IL-4), IL-5, IL-9, IL-13, and amphiregulin (Areg), by type 2 innate lymphoid cells (ILC2s) is indispensable for homeostasis, remodeling/repairing tissue structure, inflammation, and tumor immunity. Often viewed as the innate cell surrogate of T helper type 2 (Th2) cells, ILC2s not only secrete the same type 2 cytokines, but are also inextricably related to CD4^+^T cells in terms of cell origin and regulatory factors, bridging between innate and adaptive immunity. ILC2s interact with CD4^+^T cells to play a leading role in a variety of diseases through secretory factors. Here, we review the latest progress on ILC2s and CD4^+^T cells in the lung, the close relationship between the two, and their relevance in the lung disease and immunity. This literature review aids future research in pulmonary type 2 immune diseases and guides innovative treatment approaches for these diseases.

## 1. Introduction

The lung is a fragile organ, sensitive to temperature variation (cold and heat), constantly exposed to the external environment, and consequently susceptible to pathogen invasion. Thus, the lung is protected by a complex network of highly specialized immune cells and their mediators to support tissue homeostasis and prevent extensive tissue damage. The resting lung is prominently immune-tolerant, despite a plethora of leukocytes including B, T, and myeloid cells (macrophages, monocytes, dendritic cell subsets, neutrophils, and eosinophils), which numbers and proportions changing dramatically during infection and inflammation.

**Figure 1:**
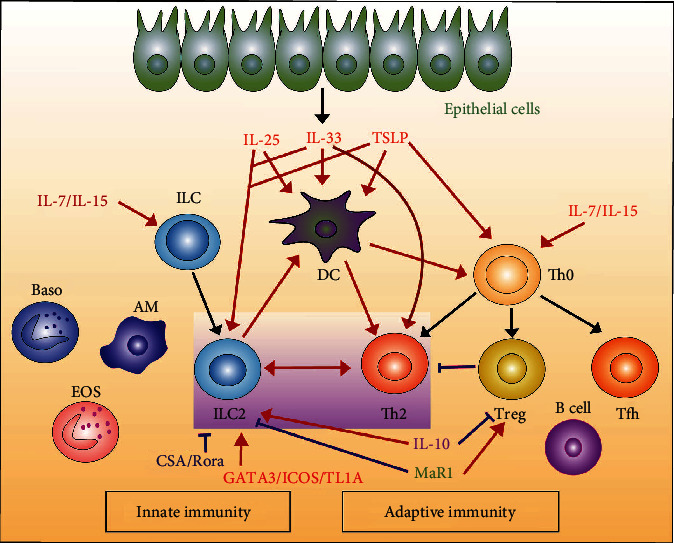
Co-stimulatory factors of ILC2s and CD4^+^T cells and their roles in innate and adaptive immunity.

Recently a small subset of other immune cells was identified in the lung, namely, innate lymphoid cells (ILCs), which are distinct from T and B lymphocytes. ILC1s, ILC2s, and ILC3s act as the innate counterparts of the traditional Th (CD4^+^T helper) 1, Th2, and Th17 T cell effector subsets, respectively [1]. Furthermore, ILCs and traditional T cells also share a common ontogeny [2–4]. Interestingly, ILC2s and Th2 cells not only share the same cytokine and transcription factor expression profile [5], but also co-regulatory signals. The homeostasis of both ILCs and T cells is supported by IL-7 and IL-15 [6, 7]. ILC2s and Th2 cells both secrete type 2 cytokines under the stimulation of epithelial factors, such as interleukin-33 (IL-33) [8] and thymic stromal lymphopoietin (TSLP) [8, 9]), and both can be activated by other factors such as GATA3 [10, 11], inducible co-stimulatory molecules (ICOS) [12], and TL1a [13, 14]. In addition to common stimulators, ILC2s and Th2 cells also share inhibitors, such as cyclosporin A (CSA) [15] and Rora [16]. However, the same factor may have different effects. IL-10 hinders T cell activation and Treg inhibition while increasing ILC2s levels [17]. Maresin 1 (MaR1) reduces lung inflammation in ILC2s but mediates the proliferation of Treg and the interaction between Treg and ILC2s [18]. Thus, ILC2s and CD4^+^T cells, especially their counterpart Th2 cells, share common stimulators and suppressors, as summarized in Figure 1.

Compared to CD4^+^Th cells, ILCs also have a unique and nonredundant genetic organization in terms of kinetics, fine-tuning, and spatial organization of the immune response [19]. Here, we will summarize the latest research progress on ILC2s and CD4^+^T cells in the lung and how their interaction participates in the immune regulation and lung homeostasis and will explore how their dysregulation can lead to lung diseases.

## 2. ILC2s in Lung Homeostasis

The ILC2 is a tissue-resident population and the major helper type ILCs in the steady-state lung of mice [20, 21]. During homeostatic conditions, this tissue-resident population is self-renewed in the lung parenchyma and is virtually absent from circulation [22]. Tissue microenvironment strongly shapes ILC2s phenotype, strikingly heterogeneous across different mucosa, and specialized modulators of regional immune responses [23]. Natural ILC2s (nILC2s) produce type 2 cytokines, while plastic inflammatory ILC2s (iILC2s) can produce both type 2 cytokines and IL-17 [24], which are considered as transient progenitor cells of ILCs and develop into nILC2s *in vitro* and *in vivo* [25]. Increased IL-25 levels, either through experimental administration or after worm infection, can trigger local proliferation and activation of intestinal ILC2s, the precursor of iILC2s, which can then reach the lungs through blood circulation [26]. Some iILC2s can differentiate into nILC2s and reside in the lung, while others home back to the small intestine [26], suggesting that ILC2s, just like adaptive T lymphocytes, are locally activated with distant effector function [26, 27]. Another study showed that ILC2s increase not only by local proliferation, but also by delivery from circulation to the lungs [28]. A new subgroup derived from ILC2s, the regulatory ILC (ILCreg) subgroup, was recently identified by the production of IL-10, therefore distinctive from activated IL-10^–^ILC2s and IL-10^+^Treg cells [29]. This population contracts after cessation of inflammatory stimulation *in vivo*, but can be restimulated upon new stimulus, similar to the Tl effector and memory cells [29].

Lung ILC2s are activated by the alarmins IL-33 [30], IL-25 [31], and TSLP [32] that is released from various immune and nonimmune cell types in response to different stimulation. After being activated, ILC2s mediate innate and adaptive type 2 immunity through rapid release of effector cytokines including IL-4, IL-5, IL-9, IL-13, and amphiregulin (Areg) and expression of co-stimulatory ligands that influence Th2 cells [33]. There are many other regulatory factors of lung ILC2s. Leukotriene cooperates with IL-33 to induce ILC2s during lung inflammation [34]. Cysteinyl leukotriene not only directly enhances IL-33 stimulated purified ILC2s to produce IL-5 and IL-13, but also promotes leukotriene-C4- and IL-33-co-induced-ILC2s activation and lung inflammation [35]. ILC2s can concurrently express ICOS and ICOS ligand, and ICOS: ICOSL signaling pathway promotes ILC2s function and homeostasis [36]. Finally, leukotrienes [37], miR-155 [38], and arginase 1 [39] can also act as key positive regulators of ILC2s.

However, the inherent defect of programmed death 1(PD-1) signaling in KLRG1^+^ILC2s could lead to uncontrolled proliferation and activation of these cells [40]. The innate immune response triggered by immunogenic extracellular RNA has a strong inhibitory effect on the proliferation and function of ILC2s *in vivo* [41]. Basophil recruitment is a hallmark of type 2 inflammation and can directly enhance the expression of neuropeptide neurotransmitter B receptor on ILC2s, which is an effective inhibitor of ILC2s [42], and androgen [43], endogenous neuropeptide calcitonin gene-related peptide [44, 45], prostaglandin E [46], and interferon-*γ* [47] are key negative regulators of ILC2s.

Interestingly, ILC2s are amateur antigen-presenting cells (APC) that can cooperate with dendritic cells (DCs) to maintain type 2 immune response [48]. ILC2s not only mediate neonatal lung development [49], but also play a role in lung diseases caused by developmental abnormalities, such as bronchopulmonary dysplasia [50]. The phenotypes of ILC2s in different models of airway inflammation are different, as manifested by ILC2s-specific expressed genes stimulated by house dust mites. These participate in adaptive immune regulation through the interaction of B cells and T cells, while the ILC2s stimulated by IL-33 express high levels of cytokine and proliferation-related genes [51]. This study indicates that lipid metabolism is required for pathogenic ILC2s response, and ketogenic diet is an effective intervention strategy for the treatment of airway inflammation [52]. However, the cognition of lung ILC2s characteristics and function is mostly based on mouse studies due to easy access to lung tissue, abundant inflammatory diseases models, and genetic impairment of ILC function.

In humans, ILC2s accumulate in fetal lungs at 10-fold higher levels than adult lungs [53]. Another study also detected ILC2s in fetal and adult lungs, which accounted for more of the total ILC in adult lungs [54]. A recent study with human blood, bronchoalveolar lavage fluid (BALF), and the lungs successfully identified ILC2s in human lung tissue and found a previously unrecognized human ILC2s population with new surface markers, such as CD30 and tumor necrosis factor receptor 2 (TNFR2), which may drive asthma [55]. However, none of the above studies directly prove the existence of human lung ILC2s by using multi-immunohistochemistry stain. Due to the presence of ILC2s in human blood, it cannot be excluded that ILC2s isolated from fragment of the human lung tissue came from contaminated blood. In other words, the existence of ILC2s in human lungs is based on suggestions rather than conclusive evidence. Better understanding of human lung ILC2s may come from less evasive samples such as human umbilical cord blood [53]/peripheral blood, or sputum and BALF [56–58], or humanized mice models [59]. Therefore, further study on human lung ILC2s, preferably directly, is needed for a better understanding of their characteristics and functions.

## 3. CD4^+^T Cells in Lung Homeostasis

CD4^+^T lymphocytes are a key element of adaptive immunity, and their role is to direct and enhance innate cell function [60]. Naive CD4^+^T cells proliferate and differentiate into several possible effector subpopulations, including traditional T helper effector cells (Th1, Th2, and Th17), T regulatory cells (Treg), and T follicular helper cells (Tfh) [60] that can support B cells [61]. Lung CD11b^+^DCs have a self-maturation process, promoting Th17 differentiation at partial maturation and Th2 differentiation at maturation [62]. These cells contribute to the maintenance of normal immune homeostasis in the face of changing microorganisms in the environment.

Early Th2 cells tilt susceptibility to allergies and are often seen as remnants of fetal maternal symbiosis [63]. Dopamine signals through specific dopamine receptors to promote Th2 differentiation and cooperates with IL-4 to enhance Th2 inflammation in the lungs of young rather than adult mice [64]. The risk of allergic asthma in infants is higher than that of adults because infants fail to induce TNF-*α* [65]. TNF-*α* migrating upregulate T-bet transcription factor in CD11b^+^DCs inducing IL-12 secretion which in turn prevents the Th2 cells differentiation [65]. It has also been found to be caused by underdeveloped Treg cells [66]. Additionally, neonatal BCG vaccination can inhibit allergic airway inflammation, by promoting T-regulatory immune response through enhanced expression of toll-like receptor 2 and 4 and PD-L1 on DC [67].

Fasting can inhibit the proliferation of Th2 cells in the lung and downregulate the level of Th2 cytokines [68]. The percentage of Th17.1, Tfh, and Tfh2 was higher in severe allergic asthma patients, while the percentage of Breg cells and Treg was significantly lower than in the control group [69]. This immune imbalance was restored after omalizumab treatment [69]. Anti-F4/80 treatment of OVA-induced asthmatic mice inhibited alternately activated macrophages and also attenuated Th2 cell response in lung tissue [70]. Similarly, reducing the levels of IL-4, IL-5, and IL-13 in the lung and the number of Th2 cells in mediastinal lymph nodes, while increasing the number of Treg cells in mediastinal lymph nodes, can alleviate allergic airway inflammation [71]. Acupuncture also seems to reduce airway inflammation and airway hyperreactivity (AHR) in asthmatic patients by correcting the imbalance of CD4^+^T lymphocyte subsets (Th1/Th2 and Treg/Th17) [72].

Fluoxetine can inhibit cancer cell proliferation in cancer patients with depression [73]. Fluoxetine increases CD4^+^Th cells while decreasing CD25^+^FOXP3^+^Treg. Additionally, fluoxetine promoted Th differentiation to Th1 cells while inhibiting Th2 and Th17 differentiation [73]. Major depressive disorder promotes the production of Th2- and Th17-related cytokines in patients with allergic rhinitis and asthma, which could be inhibited by 5-hydroxytryptamine treatment. Mechanistically, IL-5 and IL-17 levels are strongly correlated with the severity of depressive and anxiety symptoms [74].

## 4. Crosstalk between ILC2s and CD4^+^T Cells

**Figure 2:**
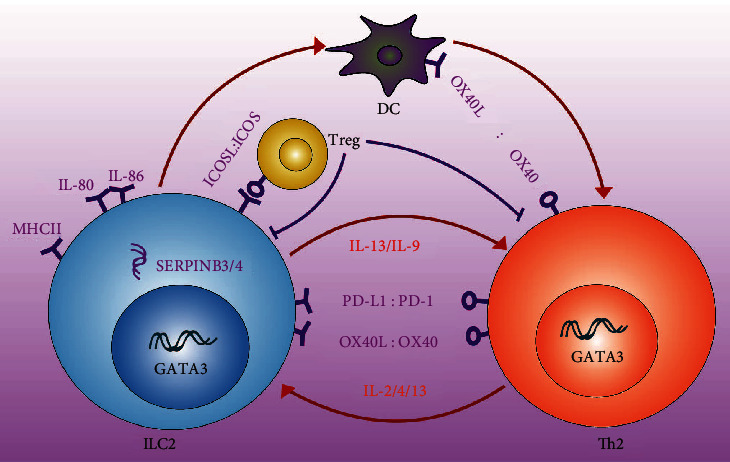
Crosstalk between ILC2s and CD4^+^T cells during type 2 immune response.

There are two main mechanisms of crosstalk between ILC2s and CD4+T cells (Table 1). During the transition to adaptive T cell-mediated immunity, a closed loop is formed between ILC2s and CD4^+^T cells, and crosstalk between the two contributes to their mutual maintenance, expansion, and cytokine production [75, 76], as summarized in Figure 2.

## 5. Crosstalk between ILC2s and CD4^+^T Cells in Lung Diseases

**Figure 3:**
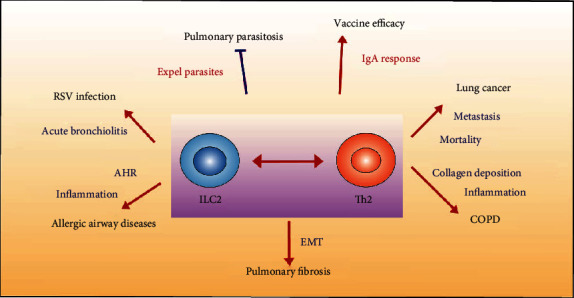
The relevance of ILC2s and Th2 cells crosstalk in lung disease.

The crosstalk between ILC2s and CD4^+^T cells, particularly with Th2 cells, plays an important role in a variety of lung diseases (Figure 3).

### 5.1. Pulmonary Parasitosis

Several parasites spread to other parts of the human body via blood circulation and often reside in the lungs (pulmonary parasitosis) causing pathological changes [87]. During helminth infection, ILC2s play a protective role by secreting IL-13 [75]. In the absence of ILC2s, Th2 cell response is impaired, which no longer produce IL-2 required for ILC2s proliferation and IL-13 production [75]. This feedback is crucial during helminth immunity and essential for helminth clearance. Thus, ILC2s with conditional deletion of PD-L1 inhibit early Th2 polarization and cytokine production, resulting in delayed excretion of helminth during infection with gastrointestinal helminth [83].

Gastrointestinal helminth infection can cause local proliferation and accumulation of ILC2s in the lungs, which is promoted by IL-4/IL-13 secreted by Th2 cells [82]. This occurs during acute type 2 immune response and is inseparable from the fact that signal transducers and activators of transcription 6 promotes the communication between Th2 cells and ILC2s in an antigen-dependent manner [82]. IL-33 mediates the activation of ILC2s and Treg cells in tissues after worm infection, and the Treg cell accumulation *in vivo* required ILC2s activation, which was independent of ILC2s secreted cytokines but partially dependent on direct co-stimulatory interactions via ICOSL:ICOS [88]. IFN-*γ* inhibits the activation of ILC2s and aggregation of Treg cells by IL-33 in infected tissues, and this inhibitory effect is enhanced with age and high-fat diet-induced obesity [88].

During helminth infection, adventitial stromal cell depletion impairs the accumulation and function of lung ILC2s and Th2 cells partially dependent on adventitial stromal cells-derived IL-33, thus impairing the effect of expelling helminth [89]. After hookworm infection, CD4^+^T cells and ILC2s cooperate to quickly expel worms within 48 hours [90]. This report shows that ILC2s plays a protective role during hookworm infection, which can be maintained by CD4^+^T cells, so as to ensure the rapid activation and maintenance of IL-13-dependent M2 macrophages immunity in the lung [90]. It should be noted that these observations are restricted to mouse models, and these crosstalk mechanisms between ILC2s and CD4^+^T cells require further confirmation in human pulmonary parasitosis.

### 5.2. Respiratory Syncytial Virus (RSV) Infection

RSV infection is a major cause of bronchiolitis and pneumonia, especially in infants and young children [91]. After treatment of RSV-infected mice, a decrease in lung mucus was observed, accompanied by a significant decrease in the number of ILC2s and macrophages, and a decrease in IL-33 in bronchoalveolar lavage fluid [92]. Similarly, during RSV infection, Th2 cell deletion can inhibit ILC2s activation and secretion of type 2 cytokines, to reduce the severity of the disease [76]. RSV infection can expand and activate CD4^+^T cells in the lungs of mice [84]. Adoptive transfer of lung ILC2s can not only increase the number of CD4^+^T cells, but can also increase the cytokine production by CD4^+^T cells, which is dependent on direct contact between them [84]. NF-B/IL-33/ST2 axis mediated the level of Th2 cytokines and the number of bronchoalveolar lavage fluid cells induced by RSV [93], and NF-B/IL-33/ST2 inhibition during RSV infection alleviated acute bronchiolitis in mice [93]. Thus, in the process of RSV infection, ILC2s and CD4^+^T cells aggravate disease by promoting each other's proliferation and activation in mice.

Human primary bronchial epithelial cells infected with rhinovirus can induce IL-33, and the culture of human T cells and ILC2s with its supernatant can strongly induce type 2 cytokines [94]. Viral infection is thought to cause chronic pulmonary interstitial inflammation and pulmonary fibrosis [95], which is described in detail next.

### 5.3. Allergic Airway Diseases

Allergic airway diseases are characterized by sneezing, itching, wheezing, chest tightness, airway obstruction, and hyperresponsiveness [96]. After allergen exposure, the number of CD4^+^T cells and ILC2s in the lung increases dramatically, with the significant increase of IL-33 expression in the lung [97]. ILC2s and CD4^+^T cells (rather than each cell population) can induce strong airway inflammation and antigen-specific type 2 immune response [97]. IL-5 and IL-13 in BALF and lung tissue mainly come from ILC2s and Th2 cells [98]. Thus, strengthening GATA3 expression is sufficient to increase the susceptibility to allergic airway inflammation by enhancing Th2 and ILC2s activity [98]. In the alveolar phase, house dust mites exposure also leads to a clear increase of IL-33, which promotes the production of cytokines in ILC2s and activates DCs, thereby promoting Th2 cell tilt [63]. IL-9 secreted by ILC2s has a similar effect [79].

Both T cells and ILC2s contribute to the deterioration of influenza-induced allergic airway inflammation, but the dynamics are different [99]. Specifically, ILC2s secreted less type 2 cytokines in the early stage of influenza-induced acute exacerbation, and became the main source after virus clearance, while T cells showed increased production of IL-4 and IL-5 in the early stage [99]. It is easy to sensitize and aggravate the degree of asthma by promoting ILC2s amplification and Th2 differentiation [100]. Blocking T cell activation and Treg inhibition and promoting the increase of ILC2s could lead to severe Th2 immune response and airway inflammation [17]. Repeated exposure to antigen preferentially triggered the increase of Ag-specific CD4^+^Th2 cells, which synergized with ILC2s, resulting in the deterioration of murine allergic airway diseases with prominent eosinophilia [101].

Thus, by reducing the number of ILC2s or CD4^+^T cells, the effect of inhibiting both can be achieved, to reduce the pulmonary inflammation of asthmatic mice [15, 102, 103]. In mice with allergic airway inflammation treated with anti-CD127 monoclonal antibody, airway resistance was significantly reduced, and lung histology was improved, accompanied by significant reduction of Th2 cytokines (IL-4, IL-5, and IL-13) in lung tissue and BALF. This also leads to a reduction of total leukocytes and specific leukocyte subsets in BALF and lung tissue, such as eosinophils, macrophages, lymphocytes, T lymphocytes, and ILC2s [104]. The regulatory DC marker C1q is as effective as dexamethasone-reducing AHR, eosinophil, and ILC2s infiltration in BALF, as well as allergen-specific Th2 cells in lung [105]. As an important regulatory cluster in T cells, Treg cells play a unique role in ILC2s. In the asthma model, the induced peripheral expansion of Treg cells effectively inhibits the proliferation of ILC2s, which may become a promising target for treatment [86].

Similar findings have been found in human. A prospective study found that Th2 cells were high during asthma exacerbation and returned to baseline levels (similar to those of stable asthma patients) after aggressive treatment, suggesting that Th2 cells may be a biomarker of impending exacerbation in asthma patients [106]. Pediatric severe asthma with fungal sensitization resulted in an increase in the number of IL-33-mediated ILC2s, Th2 cells, and steroid-resistant AHR [107]. Recently, it has been found that human umbilical cord blood-derived mesenchymal stem cells can also reduce lung type 2 (Th2 and ILC2s) inflammation [108]. Glucocorticoid is an effective drug for the treatment of asthma [109]. One study has shown that IL-13^+^ILC2s are more resistant to this therapy in humans than Th2 cells and are closely related to asthma control status [109]. Interestingly, retinoic acid stimulates human ILC2s to secrete IL-10, and this cell population is named ILCregs, which inhibit the activation of CD4^+^T cells and ILC2s [110].

However, the number of ILC2s was only found to be statistically significant in the acute exacerbation state versus the stable state, and not in the acute exacerbation phase versus after treatment in one clinical study [106]. In this study, the absolute number or percentage of Th2 cells in CD4^+^T was statistically significant in the stable state, in the acute exacerbation state, and after treatment, suggesting that Th2 cells can be used as a biomarker for acute exacerbations of asthma [106]. The inconsistency between clinical findings and basic studies regarding the role of ILC2s in asthma may be explained by the insufficient sample size of the clinical study, which means that larger longitudinal prospective studies are needed.

### 5.4. Pulmonary Fibrosis

Pulmonary fibrosis is the end-stage change in a large group of lung diseases characterized by damaged alveolar structure, the massive proliferation of fibroblasts, and deposition of extracellular matrix [111], which means that normal alveolar tissue is damaged and then abnormally repaired resulting in structural abnormalities (scar formation). Basic research confirmed that IL-13 is an important cytokine for epithelial mesenchymal transition (EMT) to promote pulmonary fibrosis [95]. Therefore, dexamethasone or anti-IL-13 can delay the progress of pulmonary fibrosis by preventing the progress of EMT[95]. In the lungs of WT mice, the frequency of IL-13^+^ILC2s peaked on day 7, and the expression of such cells was approximately five times higher than IL-13^+^CD4^+^T cells [112]. This study in a pulmonary fibrosis mice model found that ILC2s secreted IL-13 in an IL-25-dependent manner, which was independent of the antigen-specific immune responses mediated by CD4^+^T cells [112]. Areg produced by IL-33-activated-Th2 cells guides eosinophils to develop airway fibrosis both in mice and human [113, 114], and human lung ILC2s also produce Areg [115], which as a driver of tissue fibrosis [116].

### 5.5. Chronic Obstructive Pulmonary Disease (COPD)

COPD is an obstructive and progressive airway disease [117], which is characterized by severe chronic airway epithelial inflammation, leading to airway remodeling [118]. In COPD experimental model mice, T/B lymphocytes and ILC2s play a significant role in airway collagen deposition and fibrosis, but do not affect inflammation [119]. The consensus that cigarette smoking is a critical factor to induce and aggravate COPD has been confirmed in humans [120]. Reducing the number of CD3^+^CD4^+^T cells and regulating Th1/Th2 function can inhibit the progression of the COPD [120]. The increasing number of ILC2s during viral infection leads to the enhancement of inflammatory damage and, in some cases, the differentiation of ILC2s to pro-inflammatory ILC1s, resulting in acute exacerbation of COPD (AECOPD) [121, 122]. The proportion of Th2 and ILC2s was significantly increased in the peripheral blood of AECOPD patients [123]. In addition, ILC2s have the ability to mediate Th2 type adaptive immune responses in AECOPD by promoting Th2 cell differentiation through Notch-GATA3 signal pathway [123].

### 5.6. Lung Cancer

Lung cancer is one of the most lethal cancers in the world [124]. IL-33-driven activation of ILC2s inhibits IFN-*γ* production by natural killer cells, but not by CD4^+^ and CD8^+^ T cells, thereby suppressing natural killer cell-mediated intrinsic antitumor immunity and leading to increased cancer lung metastasis and mortality [125]. ILC2s promote lung metastasis of triple-negative breast cancer in a mouse tumor model [11]. They activate myeloid-derived suppressor cells by secreting IL-13, which may reduce the expansion of Tregs in the lungs of tumor-bearing mice, while promoting the proliferation of CD4^+^T cells and CD8^+^T cells [11]. IL-33 enhances T cell-mediated killing of tumor cells in primary and metastatic mouse lung tumors [126].

In lung cancer patients, low levels of stimulator of interferon genes (STING) are strongly associated with poor patient prognosis [127]. There was a positive correlation between STING cell levels and CD4^+^/CD8^+^T cell ratios and a negative correlation with Treg cell levels [127]. The low intratumoral T/Tregs ratio was associated with the early infiltration (3-6 months) of ILC2s in both primary and metastatic tumors, suggesting that Treg is involved in establishing lung immunosuppression together with ILC2s [127]. ILC2s express PD-1 which limited their intratumoral accumulation, proliferation, and antitumor effector functions [128–130]. This can be reversed by combining interleukin-33-driven ILC2s activation with PD-1 blockade [128]. Another study of patients with nonsmall cell lung cancer found that Th2 was the most abundant Th subgroup in cancer tissue and tumor tertiary lymphoid structure, followed by Treg cells, and Th1 which was the most frequently detected Th subgroup in patient-matched noncancer lung tissue [131]. In summary, the crosstalk between ILC2s and CD4^+^T seems to promote the occurrence of human primary lung cancer and mouse primary and metastatic lung cancer and its role in human metastatic lung cancer required further research.

### 5.7. Vaccine Efficacy

Vaccines are biological products made from various types of pathogenic microorganisms for immunization [132]. Vaccination induces a strong immune response to specific antigens, to prevent related diseases [132]. IL-25 and IL-33 cytokines, which regulate the activation and function of ILC2s, can differentially regulate the ILC spectrum at the vaccination site in a vaccine pathway dependent manner [133]. IL-25-binding protein can affect the quality and affinity of T cell immunity by affecting the level of IL-4/IL-13 at the vaccination site. The quality of vaccine specific T cell immunity can be improved by intramuscular injection [133]. Aluminum hydroxide salt has been added to inactivated vaccines as a safe and effective adjuvant to enhance vaccination efficacy [134].

It was found that IL-33 secretion induced by epithelial cells necroptosis initiates APC- and ILC2-mediated T cell activation, which facilitated alum-enhanced Ag-specific IgA antibody production [134]. IL-33-activated ILC2s may enhance vaccine efficacy in a Th2 cytokine-dependent manner by enhancing mucosal humoral immunity, especially IgA response [135]. Their role in establishing extensive protective and lasting humoral mucosal immunity against influenza suggests that it is helpful to develop a universal vaccine against a variety of influenza viruses [135]. The role of crosstalk between ILC2s and CD4^+^T in human vaccines is still lacking and urgently needs to be further studied.

## 6. Conclusions

CD4^+^T cells are the command center of the adaptive immune system, also known as the “helper” of the immune system, and ILC2s are important innate lymphoid cells that serve as a bridge between innate and adaptive immunity. As summarized in Figure 2, there is accumulating evidence suggesting that ILC2s and CD4^+^T cells interact in various ways, especially Th2 cells which seem to mutually promote each other's activation, proliferation, and function. Similar to the biological role of Th2 cells, ILC2s are involved in the defense against pulmonary parasitosis, the exacerbation of RSV infection, the aggravation of airway hyperresponsiveness during allergic diseases, the induction of lung injury, the acute exacerbation of COPD, the increase of cancer lung metastasis and mortality, and the improvement of vaccine efficacy, as summarized in Figure 3. Importantly, future studies should focus on elucidating the unique aspects of these interactions between ILC2s and their adaptive Th2 counterparts, especially in human, which will be particularly important for developing novel therapeutic strategies to specifically targeting type 2 immunity in human disease.

## Figures and Tables

**Table 1:** Crosstalk mechanism between ILC2s and CD4+T cells.

Effects of ILC2s on CD4^+^T cells	Effects of CD4^+^T cells on ILC2s
CD80 and CD86 on ILC2s [75]	Acting on major histocompatibility complex class II (MHCII) expressed on ILC2s [75]
IL-13 [77, 78] and IL-9 [79] secreted by ILC2s	
Regulation DCs to promote Th2 polarization [48, 80, 81]	IL-4/IL-13 secreted by CD4^+^T [82]
Contact through PD-L1:PD-1 [83] and OX40L:OX40 [84]	IL-2 secreted by CD4^+^T [76]
High mRNA expression of serine protease inhibitor B3 and B4 mRNA [85]	Contact through ICOSL:ICOS [86]

## Data Availability

The data used to support the findings of this study are available from the corresponding author upon request.
